# Optimization of the “*in‐silico*” mate‐pair method improves contiguity and accuracy of genome assembly

**DOI:** 10.1002/ece3.9745

**Published:** 2023-01-11

**Authors:** Tao Zhou, Liang Lu, Chenhong Li

**Affiliations:** ^1^ Shanghai Universities Key Laboratory of Marine Animal Taxonomy and Evolution Shanghai Ocean University Shanghai China; ^2^ Shanghai Collaborative Innovation for Aquatic Animal Genetics and Breeding Shanghai Ocean University Shanghai China

**Keywords:** accuracy, contiguity, degraded DNA, genome assembly, in silico mate‐pair

## Abstract

A combination of short‐insert paired‐ended and mate‐pair libraries of large insert sizes is used as a standard method to generate genome assemblies with high contiguity. The third‐generation sequencing techniques also are used to improve the quality of assembled genomes. However, both mate‐pair libraries and the third‐generation libraries require high‐molecular‐weight DNA, making the use of these libraries inappropriate for samples with only degraded DNA. An in silico method that generates mate‐pair libraries using a reference genome was devised for the task of assembling target genomes. Although the contiguity and completeness of assembled genomes were significantly improved by this method, a high level of errors manifested in the assembly, further to which the methods for using reference genomes, was not optimized. Here, we tested different strategies for using reference genomes to generate in silico mate‐pairs. The results showed that using a closely related reference genome from the same genus was more effective than using divergent references. Conservation of in silico mate‐pairs by comparing two references and using those to guide genome assembly reduced the number of misassemblies (18.6%–46.1%) and increased the contiguity of assembled genomes (9.7%–70.7%), while maintaining gene completeness at a level that was either similar or marginally lower than that obtained via the current method. Finally, we developed a pipeline of the optimized in silico method and compared it with another reference‐guided assembler, RagTag. We found that RagTag produced longer scaffolds (17.8 Mbp vs 3.0 Mbp), but resulted in a much higher misassembly rate (85.68%) than our optimized in silico mate‐pair method. This optimized in silico pipeline developed in this study should facilitate further studies on genomics, population genetics, and conservation of endangered species.

## Graphical abstract

We tested the in silico mate‐pair method using references with different phylogenetic distances to target species. Searching conserved mate‐pairs using multiple references improved the contiguity and accuracy of genome assembly.

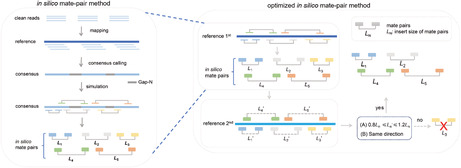

## 1 INTRODUCTION

Advances made in DNA sequencing during the past decade have led to genomes of diverse organisms being successfully sequenced and assembled (de Man et al., 2016; Iorizzo et al., 2016; Jarvis et al., 2017; Lien et al., 2016). High‐quality genome assembly requires high levels of contiguity, which enable new insights into genome structure evolution and increase the gene space completeness of the assembly (Berlin et al., 2015; Gordon et al., 2016; Koren et al., 2013; Loman et al., 2015). However, the presence of repetitive regions in a genome poses a major challenge to the assembling of highly contiguous genomes. The commonly used small insert size paired‐end data cannot span the repetitive regions, making it difficult to assemble genomes. To overcome this problem, the large insert size paired‐end sequencing, usually called mate‐pair sequencing, involves the generation of long‐insert paired‐end DNA libraries that span several kilobase pairs of long repeat regions. This is useful for many sequencing applications, including de novo sequencing, genome finishing, structural variant detection, and identification of complex genomic rearrangements (Maretty et al., 2017; Smadbeck et al., 2018; Tan et al., 2020; van Heesch et al., 2013; Wetzel et al., 2011). During mate‐pair library preparation, DNA is fragmented allowing DNA of a desired length to be isolated. Afterward, the ends of the DNA fragments are biotinylated and circularized. Then, the DNA ring is sheared into smaller fragments (400–600 bp). Biotinylated fragments are enriched (by biotin tag), and adapters ligated. These are then ready for cluster generation and sequencing. Although this technology does not produce long reads, it is able to span repeat regions if the insert size is sufficiently large. Combining data generated from mate‐pair library sequencing with those from short‐insert paired‐end reads provides a powerful combination of read lengths for maximal sequencing coverage across the genome, leading to a dramatic improvement in the assembly of large genomes. Mate‐pairs with small (1–3 kb), medium(5–15 kb), and large insert sizes (20–25 kb) are usually used to scaffold contigs in order to improve genome assemblies (Pop et al., 2004).

Third‐generation long‐read sequencing technologies, such as PacBio (Rhoads & Au, 2015) and Nanopore, (Jain et al., 2016), increase read lengths (10–100 kb) to overcome the challenge of sequencing repetitive regions that reads must be long enough to anchor in nonrepetitive sequences and span across the repeats. Repeats may be spanned, and subsequent assembling of the region is possible if the read length is substantially longer than the repeat region (Bongartz, 2019). Third‐generation long reads are also used for scaffolding during genome assembly (Boetzer & Pirovano, 2014).

High‐quality DNA, which is crucial for mate‐pair sequencing, can only be obtained from material that is both fresh and abundant. Similarly, high‐molecular‐weight DNA (>50 kb) is needed to realize the full beneficial effects of potential third‐generation sequencing. The lack of suitable starting material limits the choice of sequencing technology and affects the quality of the obtained data. For example, in a comparative genomics study of ruminants, the genomes of several species, such as mountain nyala, common eland, bongo, and oribi, were assembled at the contig level due to degenerate DNA samples, which were not suitable for constructing mate‐pair libraries (Chen et al., 2019). Another example of poor DNA involves studies of ancient DNA (aDNA; Stoneking & Krause, 2011), which mostly contains very short fragments between 44 and 172 bp (Sawyer et al., 2012).

Although it is impossible to generate libraries needed for mate‐pair or third‐generation sequencing using degenerate or ancient samples, Grau et al. (2018) invented a method that generates in silico mate‐pair libraries using a reference genome from a closely related species, thereby helping to assemble genomes at the scaffold level. In order to improve genome contiguity, they developed cross‐species scaffolding—a new pipeline that imports long‐range distance information directly into a de novo assembly process by constructing mate‐pair libraries in silico. After processing, cleaned reads of target species were mapped to the repeat‐masked reference genome, and consensus is computed. Next, read pairs of mate‐pair libraries are generated based on consensus. Finally, the cleaned reads and in silico mate‐pairs are used to assemble the genome using SOAPdenovo2 (Luo et al., 2012). Application of this in silico mate‐pair method resulted in a dramatic improvement in contiguity and accuracy, as demonstrated by the assembling of two primate genomes, based on just ∼30× coverage of shotgun sequencing data (Grau et al., 2018). A drawback of this approach is the introduction of assembly chimeras. Furthermore, phylogenetic distance, quality, and completeness of the reference genome, as well as its overall synteny and transposable element content, influence the final number of misassemblies. Methods via which misassemblies can be reduced and best references can be chosen to generate in silico mate‐pairs are yet to be tested.

In addition to the in silico mate‐pair method, referred to as the reference‐guided approach, similarity between the target and reference species can also be used to gain additional information, which often leads to more complete and improved genome assemblies (Bao et al., 2014; Pop et al., 2004; Schneeberger et al., 2011). In contrast to the in silico method that generates mate‐pairs prior to genome assembly, other reference guide approaches, such as Chromosomer (Tamazian et al., 2016), Ragout (Kolmogorov et al., 2014), and RaGOO (Alonge et al., 2019), use a single reference to order, orientate, and join contigs and long reads. However, the in silico mate‐pair method is more flexible than the reference guide approach. For example, high‐quality, conserved mate‐pairs can be selected by comparing two or more reference genomes to reduce misassemblies in the target genome assembly.

In this study, we attempted to optimize the use of the in silico mate‐pair method. First, we investigated how the phylogenetic distance between a reference and a target affects the quality of genome assembly. We then tested whether generating conserved mate‐pairs by comparing multiple reference genomes improves the quality of genome assembly. Finally, we tested the effect of the optimized in silico mate‐pair strategy on degraded samples on a simulated ancient DNA data.

## 2 MATERIALS AND METHODS

### 2.1 Experimental design

**FIGURE 1:**
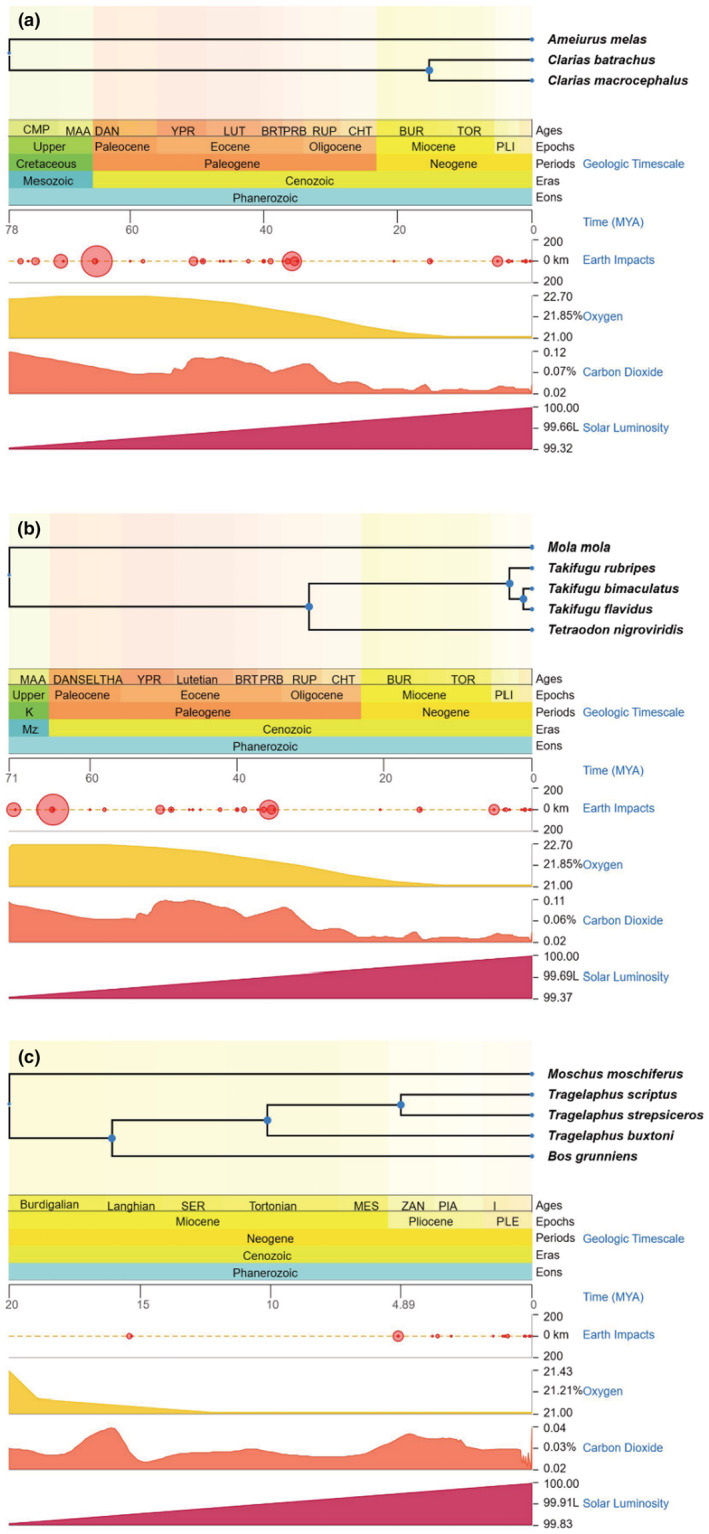
Infering the time tree from timetree.org. For taget species, *Clarias batrachus*, *Takifugu bimaculatus*, *Tragelaphus buxtoni*, species of reference genomes to generate *in silio* mate‐pairs from the same genus, family, and order were selected to infer the divergency time.

**FIGURE 2:**
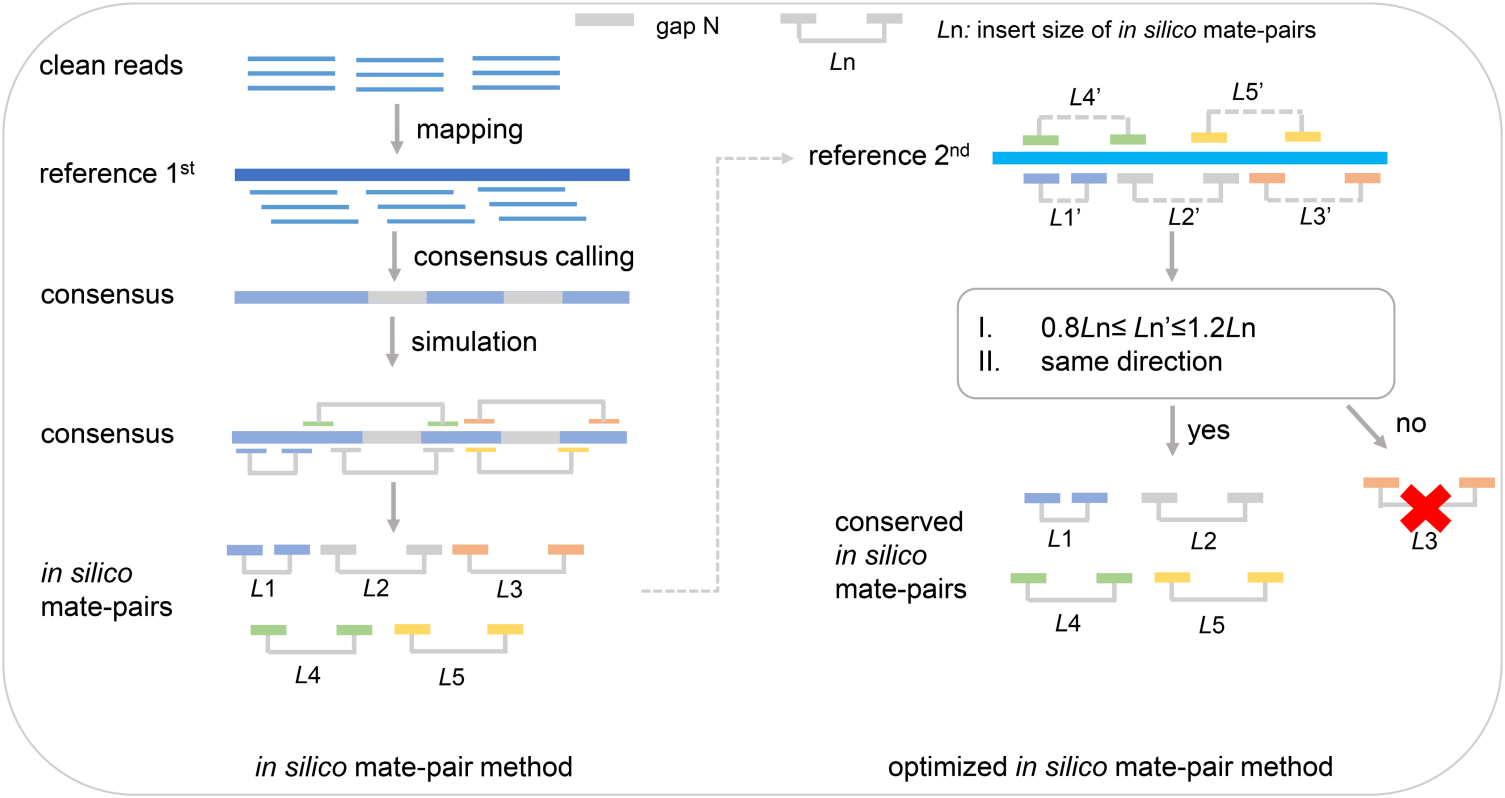
Scheme for the in silico mate‐pair method generating in silico mate‐pairs with different insert sizes using one reference genome. *L*_1_, *L*_2_, *L*_3_, *L*_4_, *L*_5_…*L*_N_ represent the different insert sizes of mate‐pairs. Optimization of *in silico* mate‐pair method (map method). Mate‐pairs generated from the first (1st) reference were mapped to the second (2nd) reference. Only mapped mate‐pairs that satisfied the insert size of “0.8 *L*n ≤ *L*n’ ≤ 1.2 *L*n” and that were also in the same direction were reserved for the following scaffolding process during genome assembly.

We designed three experiments using published data and simulations to test the efficiency of the in silico mate‐pair method and optimized in silico mate‐pair method on the genome assembly of fishes and mammals. Mate‐pair libraries were generated using multiple reference genomes with different divergency time (inferred from TimeTree, Figure 1) from the same genus, family, and order of target species (Table 1, Table S1; Kumar et al., 2022). First, we tested the effect of using references with different phylogenetic distances (Figure 1a,b) to target species, on the quality of target genome assemblies, using the paired‐end data of the walking catfish (*Clarias batrachus*) and a puffer fish (*Takifugu bimaculatus*). For *C. batrachus*, genomes of two species, *C. magur* and *C. macrocephalus*, from the same genus, and one species, *Ameiurus melas*, from a different family but the same order, were selected as references. For *T. bimaculatus*, reference genomes of two species, *T. rubripes* and *T. flavidus* from the same genus, one species, *Tetraodon nigroviridis*, from a different genus but the same family, and one species, *Mola mola*, from a different family but the same order, were selected. Second, we optimized the in silico mate‐pair method by searching for conserved mate‐pairs generated using two or more references (Figure 2) and used them to assemble the genomes via SOAPdenovo2 (Luo et al., 2012). Third, we tested whether the optimized in silico mate‐pair method using references with different phylogenetic distances (Figure 1c) significantly improved the genome assembly of the mountain nyala (*Tragelaphus buxtoni*), a highly degraded sample. Genomes of two species, *T. scriptus* and *T. strepsiceros*, from the same genus, one species, *Bos grunniens*, from a different genus but the same family, and one species, *Moschus moschiferus*, from a different family but the same order, were selected as references to produce in silico mate‐pairs for the purpose of assembling the genome of *T. buxtoni*. Lastly, we simulated single‐end ancient DNA reads using *T. flavidus* sequencing data to test the optimized in silico method and compared it with a reference‐guided approach, RagTag (Michael Alonge et al., 2022).

**TABLE 1:** Statistics of reference genomes for target species

Target species	Reference genomes	Scaffold N50	Contig N50	Number of contigs
*Clarias batrachus*	*Clarias magur*	1,316,675	1,226,249	3770
	*Clarias macrocephalus*	80,802	47,837	44,869
	*Ameiurus melas*	32,284,220	7,408,031	806
*Takifugu bimaculatus*	*Takifugu rubripes*	16,705,553	3,136,617	530
	*Takifugu flavidus*	15,676,631	4,357,567	1111
	*Tetraodon nigroviridis*	734,039	29,054	41,566
	*Mola mola*	8,766,736	23,239	51,826
*Tragelaphus buxtoni*	*Tragelaphus scriptus*	890,554	28,350	373,810
	*Tragelaphus strepsiceros*	511,483	33,649	525,298
	*Bos grunniens*	114,386,978	44,716,738	1060
	*Moschus moschiferus*	11,728,851	34,785	1,195,517

*Note*: Reference genomes from the same genus, family, and order of target species were chosen to generate in silico and optimized in silico mate‐pairs. Scaffold N50, contig N50, and number of contigs correspond to reference genomes were listed in this table.

### 2.2 Data for the target species and references

Raw data of the target species, *C. batrachus*, *T. bimaculatus, T. flavidus*, and *T. buxtoni*, were downloaded from the ENA database website (https://www.ebi.ac.uk/ena/browser/home). PCR duplicates were deleted using Prinseq (Schmieder & Edwards, 2011). Adapters and low‐quality bases were removed using Trim Galore (https://github.com/FelixKrueger/TrimGalore). Next, the reads were corrected using k‐mers with BFC (Li, 2015). Multiplicity distribution of the 23‐mers was counted using Jellyfish2 (Marçais & Kingsford, 2011) and genome coverage was estimated using KrATER (https://github.com/mahajrod/KrATER). After processing, the final genome coverage of *C. batrachus*, *T. bimaculatus*, *T. buxtoni*, and simulated ancient DNA clean reads were all more than 30 x (Table S2). The insert sizes of paired‐end reads were 180 , 300, 250, 350 bp, for *C. batrachus*, *T. bimaculatus*, *T. flavidus*, and *T. buxtoni*, respectively.

Reference genome assemblies of *C. macrocephalus*, *A. melas*, *T. rubripes*, *T. flavidus*, *T. nigroviridis*, *T. bimaculatus*, *M. mola*, *T. scriptus*, *T. strepsiceros*, *B. grunniens*, and M. moschiferus were downloaded from the National Center for Biotechnology Information (NCBI) and some characteristics of these reference genomes were listed (Table 1, Tables S3‐S5). The repeat contents of these genomes were masked using RepeatMasker (http://repeatmasker.org/)

### 2.3 Generating in silico mate‐pair libraries using the original pipeline

Multiple sets of in silico mate‐pairs were generated using the original in silico mate‐pair pipeline “cross‐mates” (Figure 2; Grau et al., 2018). First, reads of the target organism were mapped onto the repeat‐masked reference genome using BWA‐MEM (Li, 2013) and default settings. A consensus was then computed using samtools/bcftools with the samtools legacy variant calling model (Li, 2011). Mate‐pairs were sampled from the consensus in systematic mode, that is, using exact insert sizes and sampling fragments at regularly spaced offsets, and skipping regions of coverage lower than three. For the test assemblies, in silico mate‐pairs were generated with at least 30x coverage each, with multiple insert sizes ranging from 500 bp to 200 kb (500 bp, 1 kb, 1.5 kb, 2 kb, 5 kb, 10 kb, 20 kb, 50 kb, 100 kb, 200 kb). The in silico mate‐pairs generated using reference genomes from different grades of taxonomy were named as “species name*.”

### 2.4 Optimizing the method by searching conserved in silico mate‐pairs

We used a map method to search for conserved in silico mate‐pairs (Figure 2). First, mate‐pair reads generated using the first reference were mapped to another reference with BWA‐MEM (Li, 2013) and default settings, as described above. Then, an in‐house python script (sam2fq.py) was used to select the mate‐pair reads mapped within 20 percent deviation of insert sizes and in the same direction (not reversed). To distinguish conserved mate‐pairs generated from the original in silico mate‐pair method, these were named as “species1‐species2**” using two reference genomes，“species1‐species2‐species3**” using three reference genomes, and “species1‐species2‐species3‐species4**” using four reference genomes.

### 2.5 Simulation of ancient DNA reads

To investigate the efficacy of the optimized in silico mate‐pair method in regard to genome assembly of extinct species with ancient DNA, we simulated ancient DNA reads. We chose the cleaned data of *T. flavidus* to simulate ancient DNA data because it is a high‐quality genome assembly generated using both mate‐pair sequencing and PacBio sequencing. After correction, the forward strand of paired‐end reads (insert size of 250 bp, read length 150 bp) was cut at a random length to form 80 to 100 bp single‐end reads using an in‐house python script (simulate.py). The size distribution of the simulated reads is shown (Figure S1). For simulated ancient DNA, genomes of *T. rubripes* (same genus), *T. bimaculatus* (same genus), *T. nivigroviridis* (same family), *and M. mola* (same order) were selected as references. The statistics of these references are summarized (Table S6).

### 2.6 Genome assembly

Following the pipeline of Grau et al., 2018, de novo assembly of the target species genomes with in silico paired‐ends and mate‐pair reads were performed using SOAPdenovo2 (Luo et al., 2012). First, the sparse pregraph module was applied to use paired‐end or simulated ancient DNA reads during de Brujin graph construction with the parameters, ‐g 15 ‐d 4 ‐e 4 ‐R ‐r 0, and parameter ‐M 1, during the contig phase. Second, in silico mate‐pair reads generated by the original or optimized in silico mate‐pair method were mapped to contigs. Third, unique contigs were joined to scaffolds using mapped paired‐end and mate‐pair read information. For comparison with our optimized in silico mate‐pair methods, we also used the RagTag pipeline to perform genome assembly using the simulated ancient DNA reads with the following parameters: ‐f 1000 ‐d 100,000 ‐i 0.2 ‐a 0.5 ‐s 0.5 ‐r ‐g 100 ‐m 10,000. Unlike scaffolding by SOAPdenovo, the contigs produced by SOAPdenovo were ordered and oriented using RagTag.

### 2.7 Evaluation of genome assembly

Contiguity, misassemblies, and other assembly statistics were evaluated using Quast, which provides the maximum amount of information regarding assemblies (Gurevich et al., 2013). Completeness of the assemblies was measured by searching for 3354 vertebrate orthologs in a set of protein predictions generated by Augustus, as implemented in BUSCO (Simão et al., 2015). Consistent regions between the resulting genome assembly and the published genome sequence, the best assembly based on experimental mate‐pairs or third‐generation long reads, were identified using Mummer4 (Marçais et al., 2018) and then synteny between these was visualized using R (https://www.r‐project.org/).

## 3 RESULTS

### 3.1 Number of in silico mate‐pair libraries using single or multiple references

The quantities of mate‐pair read pairs generated using multiple reference genomes from the same genus, family, and order of target species were counted (Tables S7 and S8). Referring to mate‐pairs generated for *C. batrachus*, the maximum number of total in silico mate‐pair reads was generated using *C. magur* (600 M, same genus) as a reference, and even more using the *C. macrocephalus* genome as a reference (349 M, same genus). Using *A. melas* (different genus but same order) as a reference produced the minimum number of mate‐pairs (7 M). Similar results were found for in silico mate‐pair generation of *T. bimaculatus* using different references. Using *T. rubripes* and *T. flavidus* as references produced more mate‐pairs (*T. rubripes*: 268 M, *T. flavidus*: 386 M, respectively; same genus) than using *T. nigroviridis* as a reference (10 M, same family), while using *M. mola* as a reference genome produced the minimum number of mate‐pairs (1 M, same order).

The quantities of conserved mate‐pairs generated using two references (mag_mac**: 133 M) were greater than those obtained using three references (mag_mac_mel**: 4 M; Table S7). Similar results were found for the number of conserved mate‐pairs generated for *T. bimaculatus*. Using four references (two from the same genus, one same family, and one same order) produced fewer number of mate‐pairs than using three references or two (rub‐fla‐nig‐mol**: 360 K, rub‐fla‐nig**: 7 M, rub‐fla**: 121 M; Table S8). The number of conserved in silico mate‐pair libraries with different insert sizes for different target species is shown (Tables S7–S9). The number of mate‐pairs was found to decrease with the application of more reference genomes.

### 3.2 Effects of using different in silico mate‐pairs on genome assembly of *Clarias batrachus*

The assemblies of *C. batrachus* generated using only paired‐end libraries were unsatisfactory, the NGA50 only approximating 5.5 Kb and the number of complete BUSCOs (Benchmarking Universal Single‐Copy Orthologs) 1614 (Table 2). The NGA50 is NG50 where lengths of aligned blocks are counted instead of contig lengths. That is, if a contig has a misassembly with respect to the reference, the contig is broken into smaller pieces. Both the original in silico method (mate‐pairs generated using one reference from the same genus) and the optimized in silico method (conserved mate‐pairs generated using two references from the same genus) significantly improved the genome assembly of *C. batrachus*. Compared with the original in silico mate‐pair method (using a single reference from the same genus, “mag’: *C. magur* or “mac”: *C. macrocephalus*), the optimized in silico mate‐pair method (using two reference from the same genus, “mag” and “mac”) reduced misassemblies (mag*:23,519; mac*: 25,442 vs. mag‐mac**: 14,535), and yielded a similar NGA50 (mag*: 74.5 Kb; mac*: 39.1 Kb vs. mag‐mac**: 67.3 Kb) and a similar number of complete BUSCOs (mag**:2871; mac*: 2659 vs. mag‐mac**: 2788).

**TABLE 2:** Statistics of the *Clarias batrachus* assemblies

Assembly	Scaffold N50 (bp)	NGA50 (bp)	Misassemblies	Complete BUSCOs
no_ in silico	6567	5575	7861	1614
mag*	403,205	74,513	23,519	2871
mac*	130,451	39,183	25,442	2659
mel*	283,737	8247	18,552	1756
mag‐mac**	222,724	67,354	14,535	2788
mag‐mac‐mel**	6894	5537	7671	1618

*Note*: Contiguity, accuracy, and BUSCO results of the *Clarias batrachus* assemblies using the original in silico mate‐pair method (*) and optimized in silico mate‐pair method (**). “mag” short for “*Clarias magur*,” “mac” short for “*Clarias macrocephalus*,” “mel” short for “*Ameiurus melas*.” no_in silico: without in silico mate‐pair method; *: original in silico mate‐pair method using one reference; **: optimized in silico mate‐pair method using multiple references.

Compared with the original in silico mate‐pair method, optimized in silico mate‐pair method of generating conserved mate‐pairs using three reference genomes (two from the same genus “mag”, “mac,” and one from the same order “mel”) drastically decreased misassemblies (mag*: 23,519; mac*: 25,442, mel*: 18,552 vs. mag‐mac‐mel**: 8697), but did not increase the NGA50 (mag*: 74.5 Kb; mac*: 39.1 Kb, mel*: 8.2 Kb vs. mag‐mac‐mel**: 7.0 Kb) or complete BUSCOs (mag*: 2871; mac*: 2659, mel*: 1756 vs. mag‐mac‐mel**: 1915).

We compared the mate‐pairs generated using one reference genome (*C. batrachus*) with the conserved mate‐pairs generated using two reference genomes (*C. batrachus* and *C. macrocephalus*) and found that the extra mate‐pairs in the target genome generated using one reference were mostly inverted (45.76% to 47.21%), while the remaining mate‐pairs in the target genome either displayed length deviations or were mapped to different scaffolds of the target genome (Table S11).

### 3.3 Effects of using different in silico mate‐pairs on genome assemblies of *Takifugu bimaculatus*

Assembling the genome of *T. bimaculatus*, using only the paired‐end reads, yielded a NGA50 and a complete BUSCO number of 4.7 kb and 1626, respectively (Table 3). The original in silico mate‐pair method, as well as the optimized in silico mate‐pair method, improved the genome assembly of *T. bimaculatus*, significantly. Compared with the original in silico mate‐pair method (using one reference from the same genus, “rub”: *T. rubripes* or “fla”: *T. flavidus*), the optimized in silico mate‐pair method (using two reference from the same genus, “rub” and “fla”) increased the NGA50 (rub*: 140.2 Kb; fla*: 131.4 Kb vs. rub‐fla**: 183.8 Kb) and reduced misassemblies markedly (rub*:5143; fla*: 5148 vs. rub‐fla**: 4188) with comparable number of complete BUSCOs (rub*:2358; fla*: 2366 vs. rub‐fla**: 2367).

**TABLE 3:** Statistics of the *Takifugu bimaculatus* assemblies

Assembly	Scaffold N50 (bp)	NGA50 (bp)	Misassemblies	Complete BUSCOs
no_ in silico	7103	4695	1601	1626
rub*	940,637	140,231	5143	2358
fla*	858,358	131,404	5148	2366
nig*	398,444	7277	5843	1772
mol*	104,289	4760	4132	1625
rub‐fla**	1,275,322	183,811	4188	2367
rub‐fla‐nig**	24,550	7520	2159	1842
rub‐fla‐nig_mol**	7938	5222	1796	1671

*Note*: Contiguity, accuracy, and BUSCO results of the *Takifugu bimaculatus* assemblies using the original in silico method (*) and optimized in silico method (**). “rub” short for “*Takifugu rubripes*,” “fla” short for “*Takifugu flavidus*,” “nig” short for “*Tetradon nigroviridis*,” “mol” short for “*Mola mola*.” no_ in silico: without in silico mate‐pair method; *: original in silico mate‐pair method using one reference; **: optimized in silico mate‐pair method using multiple references.

Compared with the original in silico mate‐pair method, the optimized in silico mate‐pair method which generated conserved mate‐pairs using more than two reference genomes (3 references: two from the same genus, “rub,” “fla,” and one from the same order, “nig”; 4 references: using two reference from the same genus, “rub,” “fla,” one reference from the same family, “nig,” and one reference from the same order, “mol”) drastically reduced misassemblies (rub*: 5143; fla*: 5148, nig*: 5843, mol*: 4132 vs. rub‐fla‐nig**: 2249, rub‐fla‐nig‐mol*: 1615), but failed to increase either the NGA50 (rub*: 140.2 Kb; fla*: 131.4 Kb, nig*: 7.2 Kb, mol*: 4.7 Kb vs. rub‐fla‐nig**: 13Kb, rub‐fla‐nig‐mol*: 5.3 Kb) or the number of complete BUSCOs (rub*:2358; fla*:2366, nig*:1772, mol*:1625 vs. rub‐fla‐nig**: 1937, rub‐fla‐nig‐mol**: 1678).

We compared the mate‐pairs generated using one reference genome (*T. rubripes*) with the conserved mate‐pairs generated using two reference genomes (*T. rubripes* and *T. flavidus*) and found that the extra mate‐pairs generated using one reference were mostly inverted on the target genome (60.03% to 66.62%), while the remaining mate‐pairs either had length deviation on the target genome or were mapped to different scaffolds of the target genome (Table S12).

### 3.4 Genome assemblies of mountain nyala (degenerated DNA)

Mate‐pair generation of *T. buxtoni*, using *B. grunniens* as a reference, yielded the maximum number of mate‐pairs (*B. grunniens*: 416 M) while using *M. moschiferus* produced the least number of mate‐pairs (*M. moschiferus*: 220 M). The number of mate‐pairs generated using *B. grunniens* (same subfamily) as the reference genome was greater than that using *T. scriptus* and *T. strepsiceros* (same genus) as reference genomes (*T. scriptus*: 305 M, *T. strepsiceros*: 392 M), and this may be attributed to the high quality of *B. grunniens* assembly (Tables S7–S9).

The mountain nyala (*T. buxtoni*) genome, which was generated with only paired‐end reads from the degenerate samples, was not well‐assembled (Chen et al., 2019). The quality of the draft genome generated without using in silico mate‐pair libraries was unsatisfactory (N50: 3.5 kb, complete BUSCOs: 645; Table 4). Therefore, we used the original and the optimized in silico mate‐pair method to perform genome assembly of the mountain nyala. The results showed that when the original mate‐pairs were generated using different references (“scr”: *Tragelaphus scriptus*, “str”: *Tragelaphus strepsiceros*, “gru”: *Bos grunniens*, “mos”: *Moschus moschiferus*), the draft genomes were improved, showing higher contiguity (N50‐‐scr*: 592 kb, str*:431 kb, gru*:2.6 M, mos*:1.5 M) and increased completeness (Complete BUSCOs: scr*:1956, str*:1979, gru*:2018, mos*:1697). Compared with assemblies using the in silico mate‐pair method, genomes assembled using conserved mate‐pairs did not increase N50 (scr‐str**: 203 Kb, gru‐scr**: 474 Kb) or the number of complete BUSCOs (scr‐str**: 1727, gru‐scr**: 1759). Due to the low quality of the mountain nayala genome, no good reference genome could be used to calculate the misassembly rate.

**TABLE 4:** Statistics of the *Tragelaphus buxtoni* assemblies

Assembly	Scaffold N50 (bp)	Complete BUSCOs
no_ in silico	3561	645
scr*	592,242	1956
str*	431,994	1979
gru*	2,645,570	2018
mos*	1,518,369	1697
scr‐str**	203,073	1727
gru‐scr**	474,151	1759

*Note*: Contiguity and BUSCO results of the *Tragelaphus buxtoni* assemblies using the original in silico method (*) and optimized in silico method (**). “scr” short for “*Tragelaphus scriptus*,” “str” short for “*Tragelaphus strepsiceros*,” “gru” short for “*Bos grunniens*,” “mos” short for “*Moschus moschiferus*.” no_ in silico: without in silico mate‐pair method; *: original in silico mate‐pair method using one reference; **: optimized in silico mate‐pair method using multiple references.

### 3.5 Testing optimized in silico method using simulated ancient DNA reads

The quality of the genome assembly of *T. flavidus* generated using only short paired‐end libraries was unsatisfactory (N50: 0.8 kb, complete BUSCOs: 148); (Table 5). When conserved in silico mate‐pair libraries were generated using two genus references, compared with the original in silico mate‐pair libraries using one reference, the NGA50 increased (NGA50: aDNA‐rub‐bim**: 438.4 Kb vs. aDNA‐rub*:354.3 Kb), whereas misassemblies decreased significantly (misassemblies: aDNA‐rub‐bim**: 985 vs. aDNA‐rub*: 1661) and comparable numbers of complete genomes (complete BUSCOs: aDNA‐rub‐bim**: 2156 vs. aDNA‐rub*: BUSCOs: 2205).

**TABLE 5:** Statistics of the ancient DNA (*Takifugu flavidus*) assemblies

Assembly	Scaffold N50 (bp)	NGA50 (bp)	Misassemblies	Complete BUSCOs
aDNA‐no_ in silico	849	‐	1601	148
aDNA‐rub*	2,041,189	354,329	1661	2205
aDNA‐rub^@^	17,807,347	727,701	1829	2203
aDNA‐rub‐bim**	3,088,585	438,498	985	2156

*Note*: Contiguity, accuracy, and BUSCO results of the aDNA (*Takifugu flavidus*) assemblies using the original in silico method (*) and optimized in silico method (**). “rub” short for “*Takifugu rubripes*,” “bim” short for “*Takifugu bimaculatus*.” no_ in silico: without in silico mate‐pair method; *: original in silico mate‐pair method using one reference; **: optimized in silico mate‐pair method using multiple references; @: RagTag method using one reference.

Genome assembly using the RagTag pipeline showed higher contiguity (NGA50: aDNA‐rub^@^: 727.7 kb vs. aDNA‐rub‐bim**: 438.4 kb, @: assemblies using RagTag method) and higher gene completeness (complete BUSCOs: aDNA‐rub^@^: 2203 vs. aDNA‐rub‐bim**: 2156), but with many more errors (misassemblies: aDNA‐rub@: 1829 vs. aDNA‐rub‐bim**: 985), compared with using conserved in silico mate‐pair libraries generated using two genera references. Synteny between assemblies and published genome (the best assembly of *T. flavidus*) using the optimized in silico mate‐pair method performed better than that using the RagTag method on the simulated datasets with reference genomes (Figures S2 and S3).

### 3.6 Developing an automatic pipeline of optimized in silico mate‐pair method

We developed an automatic pipeline of optimized in silico mate‐pair method to process the raw reads and select the “best” reference from multiple references to generate in silico or optimized in silico mate‐pairs for assembling the genome of target species (https://github.com/TaoZhou2021/optimized‐insilico). To select the “best” reference, we first assembled the reads to contigs and scaffolds without in silico mate‐pairs using SOAPdenovo2. Then, the assembled “genome” was aligned to different reference genomes using Unimap and several characters of the corresponding alignment were calculated using an in‐house python script (optimized_insilico.py) to rank different references (1st, 2nd, 3rd, 4th, …). The original in silico mate‐pairs were generated using the 1st reference and then the conserved in silico mate‐pairs were selected using the 2nd reference. Finally, the conversed in silico mate‐pairs were used to scaffold the contigs.

## 4 DISCUSSION

High‐quality genome sequences are critical for biological research studies that focus on chromosomal structure and gene rearrangement, among others. Despite recent advances in sequencing technologies, many genome assemblies have not yet achieved the desirable level of quality. Forming the genome assemblies of some species with large or complex genomes poses challenges. Moreover, current technologies, such as long‐read sequencing and mate‐pair sequencing, cannot be used to generate high‐quality genome assemblies for some rare or extinct species, due to available DNA of these species being either degenerate or ancient. Therefore, in silico mate‐pair assembly may still be usable, especially for those species with only some degenerate DNA or ancient samples.

The phylogenetic distance to target species, quality, and completeness of the reference genome, as well as its overall synteny and transposable element content, affects the final quality of target genome assemblies. Thus, not all references are appropriate for genome assembly of a target species. Therefore, we tested multiple references with different phylogenetic distances to the genome assembly of the target species. This was demonstrated while constructing the genome assemblies of *C. batrachus*, *T. bimaculatus*, and *T. buxtoni* using in silico mate‐pair libraries that were generated using different references separately. In summary, a reference from the same genus as that of the target species is the best for making in silico mate‐pairs, compared with divergent references. In addition to phylogenetic distance, the quality of the reference genome also affected the target genome assembly. For example, the number of in silico mate‐pairs generated from the *B. grunniens* genome (different genera but same subfamily) to assemble the genome of *T. buxtoni* was higher than those generated from *T. scriptus* or *T. strepsiceros* (same genus). The genome of *B. grunniens* had an N50 of 114 Mb, which was much larger than that of *T. scriptus* (890 Kb) or *T. strepsiceros* (511 Kb). Moreover, the inferred timetree indicated an ~5MYA earlier divergency time for *T. buxtoni* than *T. scriptus* or *T. strepsiceros* though these three species are in the same genus. Nevertheless, the number of complete BUSCO genes in the target genome assembled using *B. grunniens* as the reference was only slightly higher than that using the congener as the reference. Thus, the quality and completeness of references influence the final assemblies, but to a lesser extent than the influence of the phylogenetic distance of the reference species to the target.

Misassemblies, a common issue encountered in genome assembly, are mainly caused by sequencing or assembler errors. In de novo assembly based on long sequence reads, polishing with short reads is often used to improve the base‐pair accuracy of assemblies (Rice & Green, 2019). Misassemblies in reference‐guide genome assemblers or scaffolders are inevitable due to unknown synteny and transposable element content discrepancies between the references and target species. This issue is particularly severe for assemblers that are designed based on one reference, which limits the wider use of reference‐guide assembly algorithms or tools. Thus, the feasibility of reducing misassemblies in final genome assemblies is an important issue that needs to be explored by genomic studies. Therefore, we optimized the in silico mate‐pair method by searching for conserved in silico mate‐pairs that reduce final misassemblies, under the assumption that conserved mate‐pairs would display more consistent synteny in the target species. We found that using three or more references (family or order conserved) reduced the number of misassemblies dramatically, but only by scarifying high contiguity and the number of complete genes. However, using two references from the same genus of the target species balanced contiguity, accuracy, and gene completeness of the final assemblies. By contrast, the original in silico mate‐pair method using one reference resulted in more complete genes and in more misassemblies.

An increasing amount of sequence data of aDNA samples has been observed since the initial application of high‐throughput sequencing to ancient human remains, (Rasmussen et al., 2010) over 2000 ancient samples being recorded (Brunson & Reich, 2019). In addition to the limitations of aDNA sequences, such as read length and contamination, data processing and analysis algorithms lag behind current speeds and costs. This impedes paleogenomics, with particular reference to the recovery of the full nuclear genome. The genome assembly of ancient DNA data relies on the alignment of sequencing reads to a linear reference genome, leading to the selection of endogenous DNA sequences. Thus, we simulated aDNA sequences and used these for genome assembly via different methods. The results suggested that the optimized in silico mate‐pair method performed better than the use of aDNA reads alone or the original in silico mate‐pair method. It also outperformed the assembler, RagTag, in the level of accuracy.

Use of in silico mate‐pairs for scaffolding is a simple method that enables long‐range distance information from a reference genome to be incorporated into a de novo genome assembly, via the generation of in silico mate‐pair libraries. It is essentially a novel reference‐guide approach, since other chromosome scaffolders, such as Chromosomer (Tamazian et al., 2016), MeDuSa (Bosi et al., 2015), AlignGraph (Bao et al., 2014), and RaGOO (Alonge et al., 2019), exploit distance information from a genome of a closely related organism to order and extend scaffolds or contigs after the de novo assembly process. By contrast, in silico mate‐pair libraries obtain distance information prior to the assembly process and can be adapted to any genome assembler that accepts mate‐pair sequences as input. The contiguity of a genome assembly may be improved via the application of in silico methods or other reference‐guided approaches. However, some reference‐guided scaffolders rely heavily on paired‐end or long‐length read information, making these unsuitable for single‐end reads. In addition, a large proportion of these reference‐guided scaffolders are designed based only on one reference, resulting in many misassemblies in the draft genomes. Finally, all reference‐guided genome assemblers or scaffolders have limitations, where only the conserved regions between target species and references are clear, while the sequence information between the conserved regions remains unknown.

## 5 CONCLUSION

It is crucial that the in silico mate‐pair method be used to assemble genomes from samples with only short fragment DNA, especially in the case of ancient DNA samples. Multiple reference genomes were used to select conserved mate‐pair reads prior to assembling the genome. The contiguity and accuracy of genome assemblies were significantly improved. We suggest the following: (i) infer divergency time among species from TimeTree or rank different references using our pipeline to select better references; (ii) the closer the reference, the better the in silico mate‐pair method; and (iii) the optimized in silico mate‐pair method should be used if two closely related references are available. This study provides guidelines for genome assembly using references and may benefit future genomic studies.

## AUTHOR CONTRIBUTIONS

**Tao Zhou:** Conceptualization (equal); data curation (equal); formal analysis (equal); methodology (equal); software (lead); validation (lead); writing – original draft (lead); writing – review and editing (equal). **Liang Lu:** Data curation (equal); formal analysis (equal); investigation (lead); methodology (equal); visualization (equal); writing – review and editing (equal). **Chenhong Li:** Conceptualization (equal); funding acquisition (lead); project administration (lead); resources (lead); supervision (lead); writing – review and editing (lead).

## CONFLICT OF INTEREST

The authors declare no competing interests.

### OPEN RESEARCH BADGES

This article has earned Open Data, Open Materials and Preregistered Research Design badges. Data, materials and the preregistered design and analysis plan are available at [https://data.mendeley.com/datasets/kx4t6zbxyw/1].

## Supporting information


Figure S1‐S3

Data S1

Tables S1‐S12

## Data Availability

Dataset of *C. batrachus*, *T. bimaculatus*, *T. flavidus*, and *T buxtoni* were downloaded from the ENA database website (https://www.ebi.ac.uk/ena/browser/home, SRR7440020, SRR8285222, SRR7881551, SRR6913452, SRR6913453, SRR6913455). Custom scripts used for generating the results are available at GitHub (https://github.com/TaoZhou2021/optimized‐insilico). Genome assemblies of simulated aDNA and quast output using different methods in this article were upload on the database (doi:10.17632/kx4t6zbxyw.1).
