# Supplementary figures and images for: Optimization of the “in‐silico” mate‐pair method improves contiguity and accuracy of genome assembly
Source: Ecol Evol. 2023 Jan 11;13(1):e9745. doi: 10.1002/ece3.9745 (PMC9833964; doi:10.1002/ece3.9745)

Figure S1

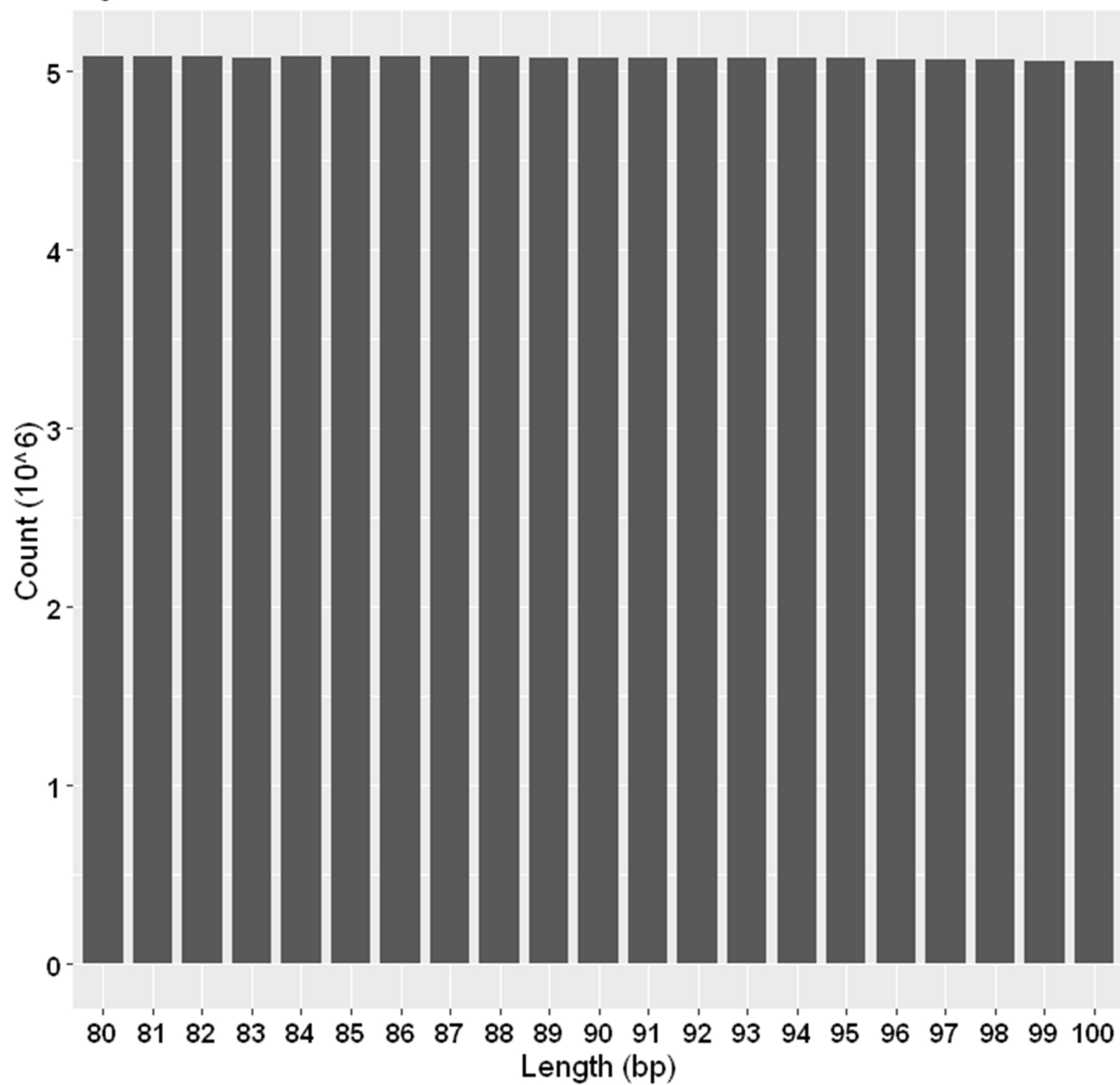

**Figure S2(A)**

Assembly

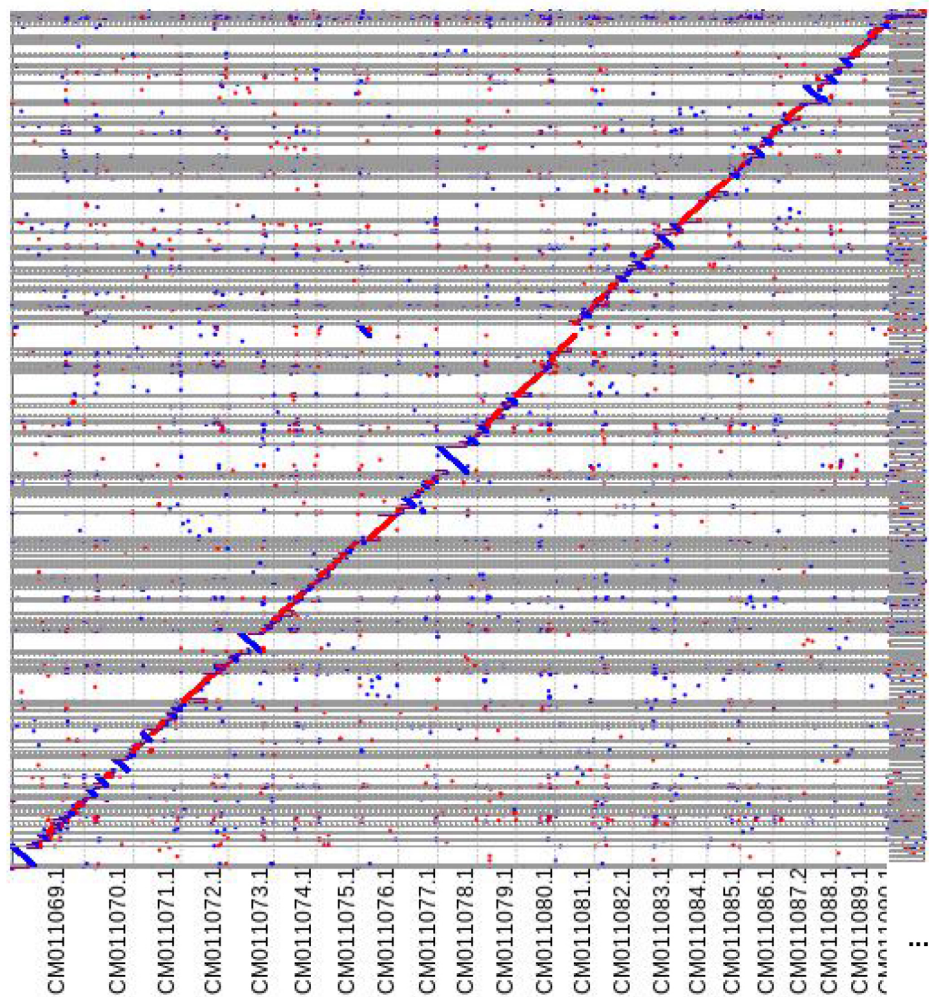

Reference

**Figure S2(B)**

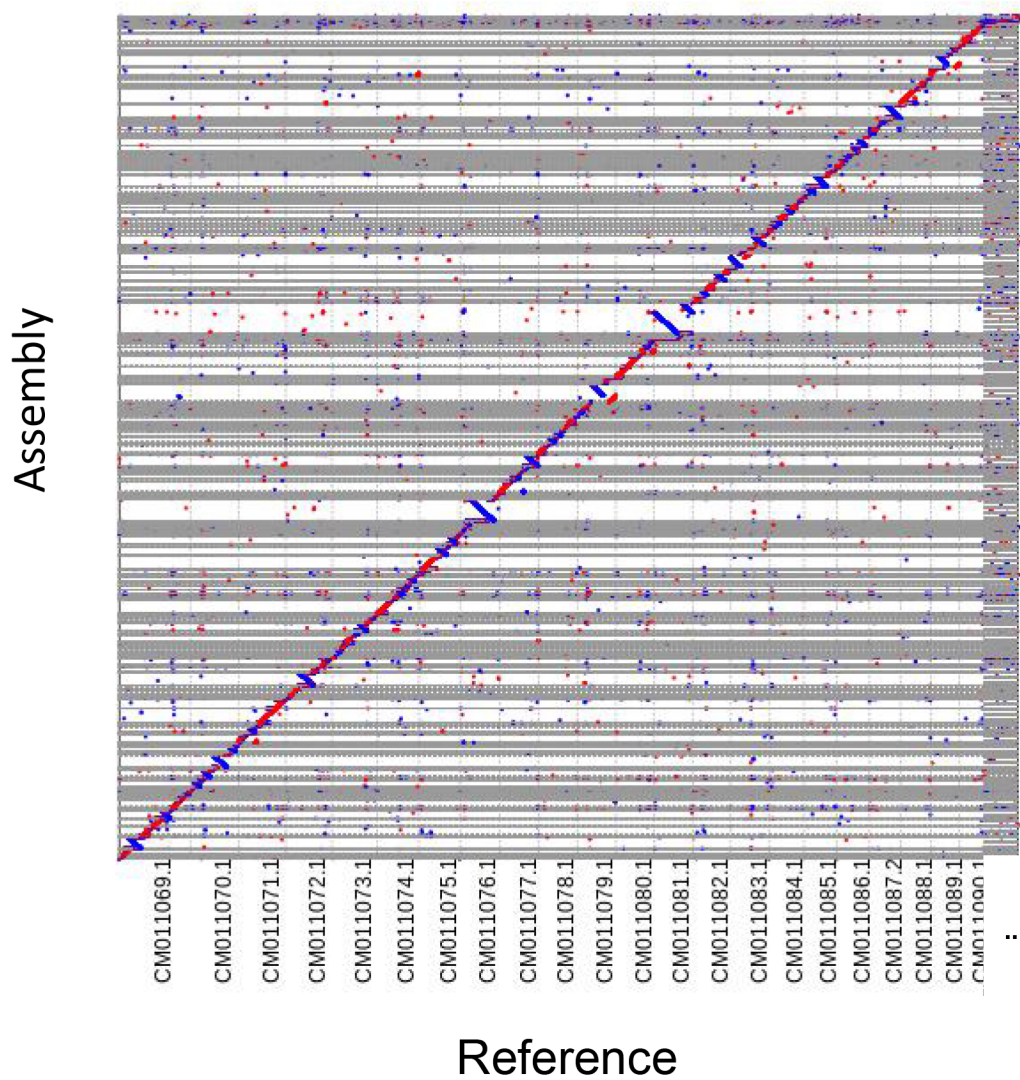

**Figure S2(C)**

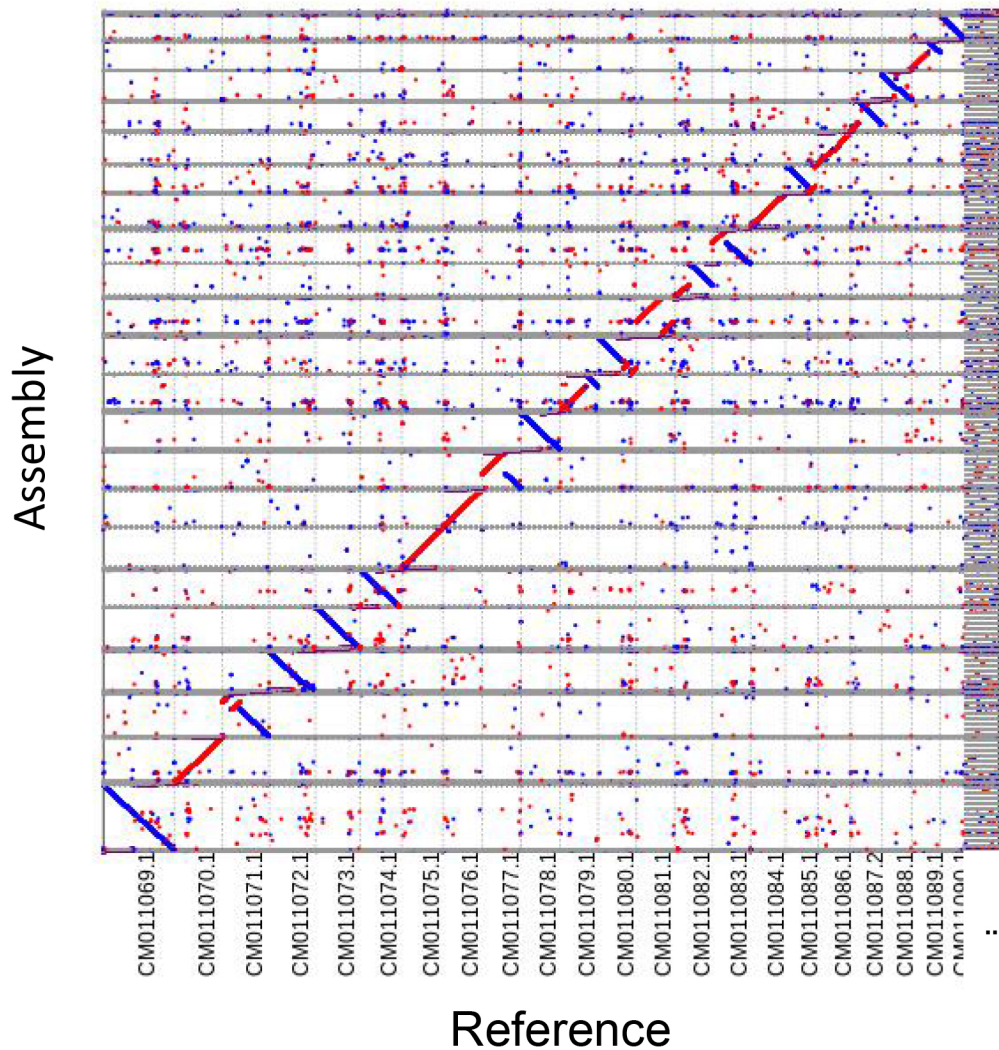

Figure S3(A)

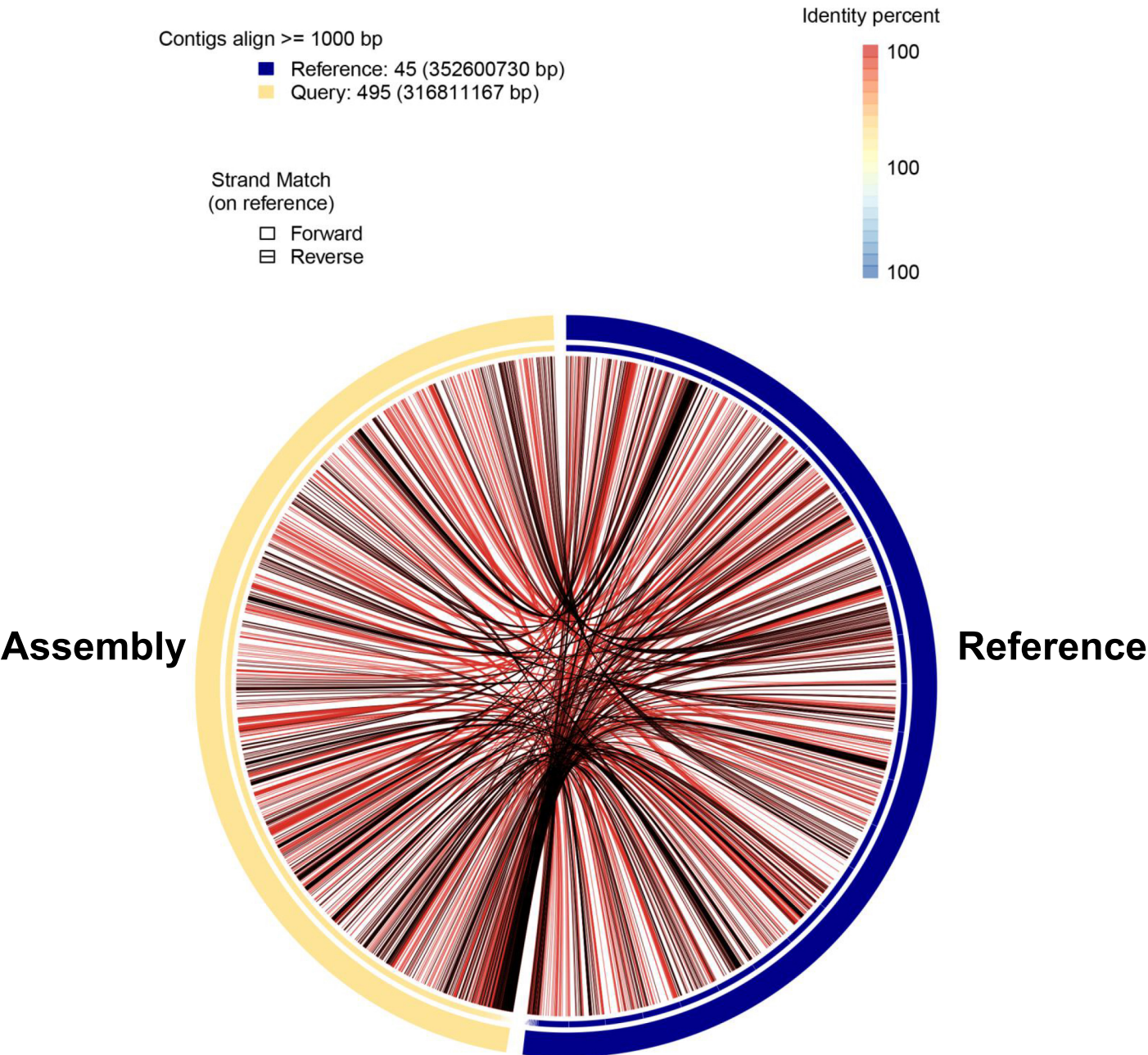

Figure S3(B)

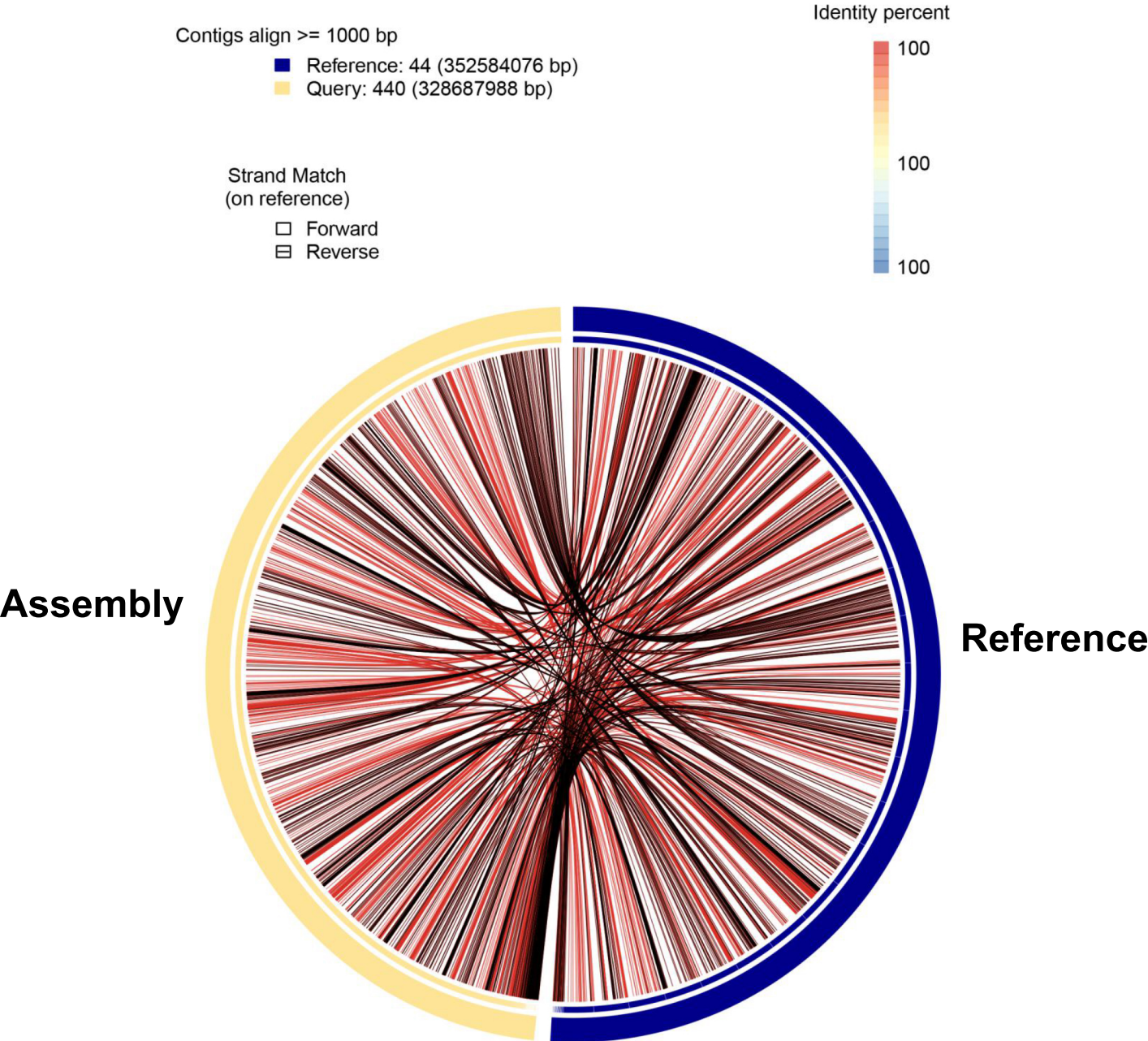

Figure S3(C)

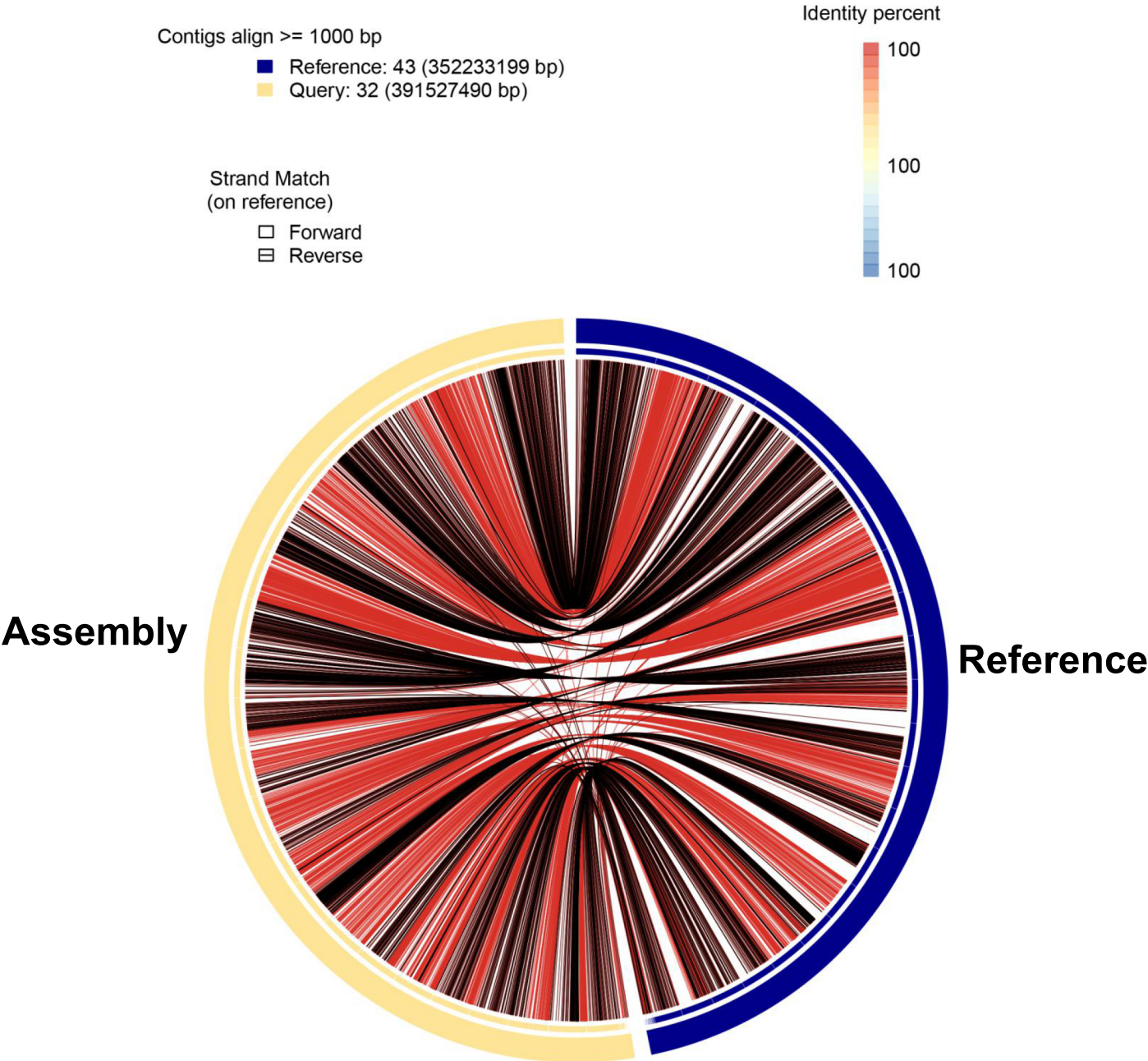

Supplement: Supplementary file 1 — Figure S1‐S3 [file ECE3-13-e9745-s002.pdf]
