# Supplementary material for: Optimization of the “in‐silico” mate‐pair method improves contiguity and accuracy of genome assembly
Source: Ecol Evol. 2023 Jan 11;13(1):e9745. doi: 10.1002/ece3.9745 (PMC9833964; doi:10.1002/ece3.9745)
Supplement: Supplementary file 2 — Data S1 [file ECE3-13-e9745-s003.pdf]

Supplemental Information for:

Optimization of the "*in-silico*" mate-pair method improved contiguity and accuracy of genome assembly

Tao Zhou[1,2], Liang Lu[1,2], Chenhong Li[1,2,*]

Supplementary Tables

The tables are included in the file: **Zhou_et_al_SupTables_20210916.xlsx**

Table S1. Statistics of reference genomes for target species

Table S2. Statistics of experimental data of target species

Table S3. Statistics of reference genomes for the *Clarias batrachus*

Table S4. Statistics of reference genomes for the *Takifugu bimaculatus*

Table S5. Statistics of reference genomes for the *Tragelaphus buxtoni*

Table S6. Statistics of reference genomes for the simulated ancient DNA (*Takifugu flavidus*)

Table S7. Numbers of mate pairs to assemble genome of the *Clarias batrachus*

Table S8. Numbers of mate pairs to assemble genome of the *Takifugu bimaculatus*

Table S9. Numbers of mate pairs to assemble genome of the *Tragelaphus buxtoni*

Table S10. Numbers of mate pairs to assemble genome of the simulated ancient DNA (*Takfifugu flavidus*)

Table S11. Align results of the extra mate pairs of the *Clarias batrachus.* (mag* vs. mag_mac**)

Table S12. Align results of the extra mate pairs of the *Takifugu bimaculatus*. (rub* vs. rub-fla**)

Supplementary Figures

The figures are included in the file: **Zhou_et_al_SupFigs_20210916.pdf**

FIGURE S1. Numbers of aDNA reads with different length simulated using clean genome data of the *Takifugu flavidus*

FIGURE S2. Synteny between assemblies (Assembly) and the best assembly of the *Takifugu flavidus* (Reference). (A) Genome assembly (aDNA-rub*) using the original *in silico* method. (B)Genome assembly (aDNA-rub-bim**) using optimized *in silico* method. (C) Genome assembly using ragoo pipeline(aDNA-rub@). aDNA: ancient DNA; rub: *Takifugu rubripes*; bim: *Takifugu bimaculatus*. *: the original *in slico* method; **: optimized *in silico* method; @: ragoo method using single reference.

FIGURE S3. Synteny comparisons of assemblies (Assembly) and the best assembly of the *Takifugu flavidus* (Reference) based on contigs align > 1000bp and 100% percent identity. (A) Genome assembly (aDNA-rub*) using the original *in silico* method. (B) Genome assembly (aDNA-rub-bim**) using optimized *in silico* method. (C) Genome assembly using ragoo pipeline(aDNA-rub@). aDNA: ancient DNA; rub: *Takifugu rubripes*; bim: *Takifugu bimaculatus*. *: the original *in slico* method; **: optimized *in silico* method; @: ragoo method using single reference.
